# The psychosocial impact of dental aesthetics and experiencing discrimination at a Peruvian public hospital among outpatients

**DOI:** 10.1371/journal.pone.0272553

**Published:** 2022-08-09

**Authors:** Claudia Carbajal, Talib Rodríguez, Diego Proaño Falconí

**Affiliations:** Carrera de Odontología, Universidad Peruana de Ciencias Aplicadas, Lima, Perú; Iowa State University, UNITED STATES

## Abstract

**Aim:**

To evaluate the association between outpatient’s perceived psychosocial impact of dental aesthetics and experiencing discrimination at a Peruvian public hospital.

**Material and methods:**

Cross-sectional study in a Peruvian public hospital, where 207 outpatients (18–30 years old) were surveyed. We asked participants about self-reported experiences of discrimination in the last six months at a Peruvian public hospital using a question from the Peruvian National Household Survey on Living Conditions and Poverty (ENAHO) 2017. We also measured the perceived psychosocial impact of dental aesthetics with the Psychosocial Impact of Dental Aesthetics Questionnaire (PIDAQ). Additionally, we evaluated age, sex, ethnicity, education, income, and reason for being discriminated against. Association was assessed with Poisson regression using a robust estimator of variance and reporting prevalence ratios with 95% confidence intervals in crude and adjusted models.

**Results:**

About two out of every five participants having experienced discrimination at a Peruvian public hospital in the last six months. On our adjusted estimates, we found discrimination to be positively associated with two components of the psychosocial impact of dental aesthetics, which were social impact (PR 1.02, 95% CI 1.00–1.04) and the psychological impact (PR 1.07, 95% CI 1.04–1.10). Conversely, dental self-confidence (PR 0.96, 95% CI 0.93–0.98) was negatively associated with discrimination.

**Conclusions:**

The perceived psychosocial impact of dental aesthetics is associated with experiencing discrimination among outpatients from a Peruvian public hospital. We advocate for structural changes to address discrimination in healthcare spaces by corresponding governmental authorities.

## Introduction

Dental aesthetics is a highly valued human feature–often associated with ideals of good health, youth, and natural beauty [1]. The perception and value of dental appearance are shaped by socio-cultural and individual ideals, which can impact a person’s psychological and social well-being positively or negatively [2]. In that sense, dental aesthetics may even become a source of discrimination [3], which is a perceived experience that “limits, deprives, and violates human rights of individuals or social groups” and may lead a person to worse health outcomes affecting their physical and mental health [4].

Three studies addressing this topic have pointed out the relevance of dental appearance on discrimination. In 2015, Moeller et al. [5] showed that having teeth with cavities, stained, broken, or missing teeth was related to discrimination among lower-income groups in Canada. They stated that those not meeting a white and aligned teeth ideal could lead to prejudice, inequality, and insecurity. Additionally, Baumgarten et al. [3] carried out a study in Brazil showing the association between dental appearance and discriminatory experiences in health services. In this study, dental appearance was classified as twisted or stained, or twisted and stained, and compared to white and aligned teeth. They found a positive association between women reporting discrimination and having twisted and stained teeth. Furthermore, Bulgarelli et al. [6] found that the absence of teeth is also related to discriminative experiences in Brazilian health care services. However, it is relevant to note that all of these studies have measured dental aesthetics clinically and have not measured the dental aesthetic perception of the person reporting discrimination. So, there is a gap of knowledge relating perceived dental aesthetics to discrimination in a health care service.

In Peru, discrimination is a relevant issue to healthcare services. The Peruvian national survey of perceptions on cultural diversity and ethnic-racial discrimination showed that people felt discrimination most commonly in public hospitals or health centers [7]. Additionally, the survey results suggest that discrimination has been normalized in Peru, as more than half of the respondents reported discrimination. Additionally, despite more than half claiming that others are racist or very racist, only one in every twelve self-claimed themselves as racist. On the other hand, one of every five Peruvians considers that the main reason for perceiving discrimination is facial or physical features [7]. Therefore, our study aimed to evaluate the association between the outpatient perceived psychosocial impact of dental aesthetics and experiencing discrimination at a Peruvian public hospital.

## Materials and methods

### Study design

Cross-sectional study conducted from August to November 2019.

### Setting, participants, and sample

The sampling was non-probabilistic by convenience. We chose to conduct this study in a referent Peruvian public hospital in the capital city, Lima. This institution (Maria Auxiliadora Hospital) provides services and is the only referral center in the southern sector of Lima and provides comprehensive health care services for 89,619 outpatients between 18–29 years old in 2019 [8].

In our study, we included patients between 18 and 30 years of age who were outside the public hospital waiting for their appointment or coming out from one. Additionally, patients were only eligible if they had visited the hospital in the last six months. We did not include those who did not wish to participate, companions, those with speaking or writing difficulties, or who were heading to the emergency department.

The sample size was calculated using the mean comparison formula (95% confidence level, 80% power, 5% alpha) with the statistical program Stata® v15.0. For that matter, we used a previous study performed in Peru that analyzed ‘Satisfaction with dental appearance’ associated with malocclusion psychosocial impact ($\bar{x}$ = 31.77, sd = 11.10) [9]. The formula yielded an estimate of 198 participants, of which 207 self-administered surveys were performed.

Our research was approved by the Ethics Committee of the Faculty of Health Sciences of the Universidad Peruana de Ciencias Aplicadas under the project code CEI PI067-19. Voluntary participation was granted by signing an informed consent form, which participants got a copy. Additionally, the dataset for this investigation has been uploaded as supplementary material (S1 Dataset).

### Measurements

#### Survey

The questionnaire was self-administered by patients. The self-reported survey consisted of seven items selected from the National Household Survey on Living Conditions and Poverty (ENAHO) 2017 that were used to respond to age, sex, discrimination, ethnicity, education, and income. Additionally, the Psychosocial Impact of Dental Aesthetics Questionnaire (PIDAQ) was employed to respond for outpatient’s perceived psychosocial impact of dental aesthetics [10].

#### Self-reported experiences of discrimination

Participants answered yes or no to the question, “In the last 6 months, have you felt mistreated, or have they tried to discriminate against you in this healthcare establishment?” The original single-item question comes from the module of governance, democracy, and transparency in the ENAHO 2017 [11]. But, we modified the following: “in the last 5 years” to “in the last 6 months”, and “in healthcare establishments (healthcare services, hospital, etc.)” to “in this health*care establishment*.*”*

#### Perceived psychosocial impact of dental aesthetics

The PIDAQ is a self-reported instrument that evaluates the perceived psychosocial impact of dental aesthetics in young adults between 18 and 30 years of age [12]. For this research, the Spanish-translated version was used, which has a general Cronbach’s α coefficient of 0.93, and of 0.87 to 0.93 in its four subscales [13]. These four subscales are dental self-confidence (six items), social impact (eight items), psychological impact (six items), and aesthetic concern (three items) [12]. Furthermore, dental self-confidence measures the positive impact of dental aesthetic on a person (e.g. I am proud of my teeth), social impact conveys a participant’s worry on the reactions others would have when looking at their teeth (e.g. I’m afraid other people could make offensive remarks about my teeth), psychological impact reflects negative feelings of unhappiness (e.g. I think most people I know have nicer teeth than I do), and aesthetic concern showed participant’s disapproval of their own dental appearance (e.g. I don’t like to see my teeth in mirrors) [12, 14].

All scorings used a Likert scale where 1 = strongly disagree and 5 = strongly agree, and where then modified so that the minimum score was 0 and maximum 4. Also, as the self-confidence component has a positive wording, scoring for this subscale was reversed. Therefore, the final PIDAQ score had a range from 0–92 and the higher the score was when summed, the greater the psychosocial impact of dental aesthetics on the respondent’s oral health-related quality of life [10, 12, 13].

#### Covariates

Participants indicated their age in years and gender (male/female). Then, questions from the ENAHO 2017 were used for ethnicity, educational level, income, and reason for being discriminated against.

The question used for ethnicity was, “Because of your customs and your ancestors, do you feel or consider yourself: Quechua, Aymara, native or Indigenous of the Amazon, belonging to or part of another Indigenous or native people, black/Moreno/Zambo/mulatto/Afro-Peruvian or Afro-descendant people, white or Mestizo?” For better statistical comparison, we re-categorized these groups into Mestizo, Quechua, and others (which included all the other ethnicities). It is relevant to note that Peruvians most commonly (around 60% of the population) self-identify themselves as Mestizo, that is, as a mixed-race individual [15].

Participants were asked about their education level (no level or initial education, primary education, secondary education, and higher education) through the question, "What is the last year or grade of studies and level that you passed?” For better statistical comparison, we merged the first three groups (secondary education or lower).

To estimate income, participants were asked, “How much was your total income [in Peruvian national currency] in the previous month, including overtime, bonuses, payment for refreshments, mobility, commissions, etc.?” We then divided these responses into quartiles from poorest to wealthiest.

Finally, we asked participants about the reason for being discriminated against “What do you think was the main reason you were treated this way?” Responses were “due to skin color/race, place of origin or residence, poverty, sexual orientation, or other.” Similar to the previous questions, due to few responses for skin color/race and sexual orientation we decided to merged them with “other.”

#### Analysis plan

All analyses were performed using the statistical program Stata® v15.0. Absolute and relative frequencies were reported for sex, ethnicity, education, income, and discrimination, while mean and standard deviations for age and PIDAQ (dental self-confidence, social impact, psychological impact, aesthetic concern, and psychosocial impact of dental aesthetics). The proportion of patients who self-reported discrimination was compared by sex, ethnicity, and income using the Chi-square test. On the other hand, age, the psychological impact, and the total score for the psychosocial impact of dental aesthetics were compared using the Student’s T-test, as these met normality criteria and variance homogeneity. Additionally, dental self-confidence, social impact, and aesthetic concern were compared with self-reported discrimination using the U Mann Whitney test because they did not meet the criteria for normality.

To analyze the association between the psychosocial impact of dental aesthetics and self-perceived discrimination prevalence ratios with a 95% confidence interval in a log-binomial regression was initially sought through a crude and adjusted model (which included sex, age, ethnicity, income, and education), but failed to converge. Alternatively, we performed a Poisson regression with a robust variance estimator (also known as sandwich estimator or modified Poisson) [16]. As suggested by Barros and Hirakata (2003) [17], modified Poisson is an alternative to logistic regressions for cross-sectional studies with binary outcomes. Moreover, it is relevant to note that the binary outcome (self-perceived discrimination) was common (38%), and model tests of goodness-of-fit (Deviance and Pearson) did not yield statistical significance under any model (p≈1.000).

## Results

Table 1 shows the general characteristics of the 207 adult outpatients (18–30 years of age) from a Peruvian public hospital in Lima. The sample comprised 23 years old adults on average, who were most commonly women, of Mestizo ethnicity, and three of every four participants had secondary or lower education. Also, about two of every five participants reported having experienced discrimination in the last six months. Participants pointed out that poverty and other reasons were the causes for being discriminated against. Lastly, average PIDAQ scores were reported for the four subcomponents and the general score.

**Table 1:** Characteristics of 207 adult outpatients from a Peruvian public hospital.

	Sample	
Variables	n	(%)
*Age in years* *	23	(3.7)
*Sex*		
Female	112	(54.1)
Male	95	(45.9)
*Ethnicity*		
Mestizo	108	(52.2)
Quechua	66	(31.9)
Other**	33	(15.9)
*Education*		
Secondary or lower	161	(77.7)
Higher	46	(22.2)
*Income*		
Q1 (*Poorest*)	57	(27.5)
Q2	53	(25.6)
Q3	52	(25.1)
Q4 (*Wealthiest*)	45	(21.7)
*Experienced discrimination*		
Yes	79	(38.2)
No	128	(61.8)
*Reason for being discriminated against*		
Place of origin or residence	22	(10.6)
Poverty	28	(13.5)
Other reasons	29	(14.0)
NA	128	(61.8)
*Dental self-confidence* *	11.5	(6.9)
*Social impact* *	13.4	(8.4)
*Psychological impact* *	11.6	(5.9)
*Aesthetic concern* *	4.4	(3.8)
*PIDAQ general score* *	64.7	(19.1)

*Mean (standard deviation)

** Aimara, Native or Indigenous to the Amazon, Belonging to or part of another Indigenous or native people, Black/Moreno/Zambo/Mulatto/Afro-Peruvian or Afro-descendant, and White.

NA = Not applicable as they did not feel discriminated.

Minimum/Maximum possible scores of dental self-confidence = 0/24; social impact = 0/32; psychological impact = 0/24; aesthetic concern = 0/12; general PIDAQ = 0/92

Table 2 shows that we did not find much differences between age, sex, ethnicity, education, income and having experienced discrimination in the past six months. Nevertheless, among the ethnic groups Quechua ethnic group most commonly reported discrimination, as well as those with a secondary or lower education. On the other hand, PIDAQ scores for those experiencing discrimination were higher on social impact, psychological impact, and slightly higher for aesthetic concern. Contrarily, dental self-confidence scores were higher among those not experiencing discrimination.

**Table 2:** Prevalence of experiencing discrimination and participant’s characteristics among 207 outpatients from a Peruvian public hospital.

	Experienced discrimination			Did not experience discrimination		
Variables	n	(%)	95%CI	n	(%)	95%CI
*Age in years* * ^,^ ^γ^	24.2	(3.5)	[23.4–25.0]	23.7	(3.8)	[23.1–24.4]
*Sex*						
Female	43	(38.4)	[29.8–47.8]	59	(62.1)	[52.2–70.2]
Male	36	(37.9)	[28.6–48.1]	69	(61.6)	[51.9–71.3]
*Ethnicity*						
Mestizo	38	(35.2)	[26.7–44.7]	70	(64.8)	[55.3–73.3]
Quechua	29	(43.9)	[32.4–56.2]	37	(56.1)	[43.8–67.6]
Other**	12	(36.4)	[21.7–54.1]	21	(63.6)	[46.0–78.3]
*Education*						
Secondary or lower	65	(40.4)	[33.0–48.2]	96	(59.6)	[51.8–67.0]
Higher	14	(30.4)	[18.8–45.3]	32	(69.6)	[54.7–81.2]
*Income*						
Q1 (*Poorest*)	19	(33.3)	[22.2–46.6]	38	(66.7)	[53.4–77.8]
Q2	22	(41.5)	[29.0–55.3]	31	(50.5)	[44.7–71.1]
Q3	20	(38.5)	[26.2–52.4]	32	(61.5)	[47.6–73.9]
Q4 (*Wealthiest*)	18	(40.0)	[26.7–55.0]	27	(60.0)	[45.0–73.3]
*Dental self-confidence* * ^,Ꞃ^	9.5	(6.7)^b^	[8.0–11.0]	12.8	(6.7)	[11.7–14.0]
*Social impact* * ^,Ꞃ^	15.2	(8.6)^a^	[13.3–17.1]	12.2	(8.1)	[10.8–13.6]
*Psychological impact* * ^,^ ^γ^	13.7	(5.2)^b^	[12.5–14.8]	10.2	(5.9)	[9.2–11.3]
*Aesthetic concern* * ^,Ꞃ^	4.9	(3.9)	[4.0–6.7]	4.1	(3.8)	[3.5–4.8]
*PIDAQ general score* * ^,^ ^γ^	43.2	(14.3)	[40.0–46.4]	39.4	(12.9)	[37.2–41.7]

*Mean (standard deviation)

** Aimara, Native or Indigenous to the Amazon, Belonging to or part of another Indigenous or native, Black/Moreno/Zambo/Mulatto/Afro-Peruvian or Afro-descendant, White.

^γ^ Student T-test; ^Ꞃ^ Mann Whitney U test; Chi-square test was used for categorical variable comparison

^a^ p<0.05

^b^ p<0.001

Minimum/Maximum possible scores of dental self-confidence = 0/24; social impact = 0/32; psychological impact = 0/24; aesthetic concern = 0/12; general PIDAQ = 0/92

Table 3 depicts the association between the perceived psychosocial impact of dental aesthetics and experiencing discrimination. Crude model estimates show a positive association for the PIDAQ general score (PR: 1.02, 95% CI 1.00–1.03), social impact (PR: 1.03, 95%CI 1.01–1.05), and psychological impact (PR: 1.07, 95%CI 1.04–1.10). Dental self-confidence, conversely, showed a negative association (PR: 0.96, 95% CI 0.93–0.98). Estimates remained similar in the adjusted model, which controlled for age, sex, ethnicity, education and income.

**Table 3:** Association between the psychosocial impact of dental aesthetics and experiencing discrimination among 207 outpatients from a Peruvian public hospital.

		Experienced discrimination			
		Crude association		Adjusted association	
PIDAQ	Units	PR^a^	[95% IC]	PR^a^^,^ ^b^	[95% IC]
*Dental self-confidence*	*Points*	0.96	[0.93–0.98]**	0.96	[0.93–0.98]**
*Social impact*	*Points*	1.03	[1.01–1.05]*	1.02	[1.00–1.04]*
*Psychological impact*	*Points*	1.07	[1.04–1.10]***	1.07	[1.04–1.10]***
*Aesthetic concern *	*Points*	1.03	[0.99–1.08]	1.03	[0.98–1.07]
*PIDAQ (general score)*	*Points*	1.02	[1.00–1.03]*	1.01	[1.00–1.02]

PIDAQ = Psychosocial Impact of Dental Aesthetics Questionnaire

*< 0.05 **<0.01 ***<0.001

^**a**^ Poisson regression with a robust variance estimator was performed and prevalence ratios (PR) are reported.

^b^ Adjusted for age, sex, ethnicity, income, education

## Discussion

We found that the perceived psychosocial impact of dental aesthetics is associated with experiencing discrimination at a Peruvian public hospital among outpatients. More specifically, for every one-point increase in the score of the psychological impact component, there is a 7% greater chance of experiencing discrimination at the public hospital. Conversely, dental self-confidence showed a negative relationship with being discriminated against, where a one-point increase was associated with 4% fewer chances of experiencing discrimination. Additionally, the prevalence of discrimination in our study sample (about two-of-every five patients) is twice as large as that reported in national estimates of Peru and the USA in healthcare services [7, 18] and more than twice as large as that reported in Brazilian healthcare services [3, 6].

Before explaining our study findings, some study limitations must be addressed. Firstly, our cross-sectional design could only permit us to test associations rather than causations. Secondly, we conducted surveys in the hospital surroundings to participants who had an appointment in the hospital in the last six months, which could mean that our findings did not reflect the participant’s experience at the time of the interview and could also induce recall bias. However, we don’t expect this to have affected our findings as six months is considered a good recall period [19] and is shorter than the two-year timeframe used in the Baumgarten et al. study [3]. Additionally, a too-short recall period may be inappropriate for infrequent events such as outpatient visits [20]. Thirdly, as a self-reported questionnaire, our study could be prone to desirability bias, and perception minimization bias [21], which could ultimately underestimate our results. Nevertheless, we found higher estimates of discrimination than in other studies in public healthcare settings [3, 6, 7, 18], and participants were reminded of confidentiality and anonymity during the survey. Fourthly, the question on discrimination included the word “mistreatment”, which participants may have felt instead of discrimination. However, “mistreatment”, “unfair treatment”, or similar wordings are commonly employed in self-reported questionnaires and national surveys on discrimination, which allows comparability [22]. Fifth, we could not single out a physical dental trait with which participants were least satisfied. Nonetheless, we used a condition-specific health-related quality of life questionnaire that provides insight into the individual’s perception of their dental aesthetics [12].

One of the explanations for the social and psychological impact of dental aesthetics to be positively associated with experiencing discrimination at a public hospital is the influence of socio-cultural aspirations and individual ideals [2, 3, 13, 23], possibly enhanced in these spaces by healthcare staff or images displayed in the building. Thus, making people concerned about others’ reactions to their teeth (social impact) or unhappiness towards their teeth compared to others (psychological impact). Furthermore, participants pointed out poverty as one of the main reasons for experiencing discrimination at the hospital. Additionally, the self-image disconnection between people and publicity images (also present in healthcare spaces) is frequent, as one-third of Peruvians have reported feeling unrepresented in publicity/commercials, and almost half of the population doesn’t feel represented in magazines [7]. Another explanation may be due to the age of the participants (18–30 years old). Because young adults are now constantly engaging in social media, which may reflect body dissatisfaction and reduced satisfaction with facial appearance, which increases with high levels of self-discrepancy [24].

On the other hand, dental self-confidence was found to be negatively associated with experiencing discrimination. We postulate that the reason is due to the positive emotional state despite one’s dental appearance may attenuate the perceived psychosocial impact of dental aesthetics when dental aesthetic ideals are not met [14]. But, it could also be the other way around, where those with high dental self-confidence usually have met their ideal dental aesthetics [25]. Additionally, this means that people with low self-confidence may feel insecure and perceive discrimination more frequently [5, 12].

We recommend that the findings provided in this research be used to prioritize a broader aspect of health care, such as structural racism [26]. We advocate for health institutions to address discrimination at healthcare centers. Firstly, it is imperative to investigate the reasons behind the high number of outpatients reporting experiences of discrimination. In our study, we identified poverty as one of those main reasons, but we did not explore more on other reasons. Secondly, healthcare centers should enable patient participation and engagement with the publicity shown, so images of their surroundings may be more relatable. Thirdly, healthcare workers should not contribute to this discrimination to avoid negatively affecting patients’ trust in the healthcare center [18]. Furthermore, future research on this topic should focus on evaluating the perceived psychosocial impact of dental aesthetics on older ages [12, 27] and studying the psychosocial impact of dental aesthetics on the private sector [7].

## Conclusions

Our study shows that the perceived psychosocial impact of dental aesthetics is associated with experiencing discrimination at a Peruvian public hospital among outpatients. We also found that about two-out-of-every-five patients experienced discrimination and pointed out that poverty was one of the main reasons for this experience. We suggest structural changes in public healthcare settings are necessary through patient participation and engagement–to address this issue and create a more friendly environment.

## Supporting information

S1 Dataset (XLSX)

## References

[1] Khalid A, Quiñonez C. Straight, white teeth as a social prerogative. Sociol Health Illn. 2015 Jun;37(5):782–96. doi: 10.1111/1467-9566.12238 25923766

[2] Afroz S, Rathi S, Rajput G, Rahman SA. Dental Esthetics and Its Impact on Psycho-Social Well-Being and Dental Self Confidence: A Campus Based Survey of North Indian University Students. J Indian Prosthodont Soc. 2013 Dec;13(4):455–60. doi: 10.1007/s13191-012-0247-1 24431775 PMC3792334

[3] Baumgarten A, Bastos JL, Toassi RFC, Hilgert JB, Hugo FN, Celeste RK. Discrimination, gender and self-reported aesthetic problems among Brazilian Adults. Community Dent Oral Epidemiol. 2018 Feb;46(1):24–9. doi: 10.1111/cdoe.12324 28737282

[4] Williams DR, Lawrence JA, Davis BA, Vu C. Understanding how discrimination can affect health. Health Serv Res. 2019 Dec;54(S2):1374–88. doi: 10.1111/1475-6773.13222 31663121 PMC6864381

[5] Moeller J, Singhal S, Al-Dajani M, Gomaa N, Quiñonez C. Assessing the relationship between dental appearance and the potential for discrimination in Ontario, Canada. SSM—Popul Health. 2015 Dec 1;1:26–31. doi: 10.1016/j.ssmph.2015.11.001 29349118 PMC5757998

[6] Bulgarelli AF, dos Santos CM, Rech RS, Baumgarten A, Goulart BN. Tooth Loss Condition and Social Discrimination in Brazilian Healthcare Services. Int J Public Health. 2021 Mar 12;66:586597. doi: 10.3389/ijph.2021.586597 34744559 PMC8565290

[7] Ministerio de Cultura. Percepciones y Actitudes sobre Diversidad Cultural y Discriminación Étnico-Racial. Lima: Ministerio de Cultura; 2018.

[8] Ministerio de Salud, Hospital María Auxiliadora, Oficina de Estadística e Informática. Compendio Estadístico 2019 [Internet]. Lima: Ministerio de Salud; 2020. Available from: https://cdn.www.gob.pe/uploads/document/file/3059702/Compendio%20Estadistico%202019.pdf

[9] Rojas H. Asociación de maloclusión y la satisfacción de la apariencia dental en adolescentes de la institución educativa privada nueva generación de Ate Vitarte 2018. [Lima]: Universidad Nacional Federico Villareal;

[10] Bucci R, Rongo R, Amato A, Martina S, D’Antò V, Valletta R. The Psychological Impact of Dental Aesthetics in Patients with Juvenile Idiopathic Arthritis Compared with Healthy Peers: A Cross-Sectional Study. Dent J. 2019 Oct 1;7(4). doi: 10.3390/dj7040098 31581530 PMC6960518

[11] Instituto Nacional de Estadística e Informática (INEI). Encuesta Nacional de Hogares 2017. Encuesta de Opinón. Módulo: Governabilidad, Democracia y Transparencia. Lima: INEI;

[12] Klages U, Claus N, Wehrbein H, Zentner A. Development of a questionnaire for assessment of the psychosocial impact of dental aesthetics in young adults. Eur J Orthod. 2006 Apr;28(2):103–11. doi: 10.1093/ejo/cji083 16257989

[13] Montiel-Company JM, Bellot-Arcís C, Almerich-Silla JM. Validation of the psychosocial impact of dental aesthetics questionnaire (Pidaq) in Spanish adolescents. Med Oral Patol Oral Cir Bucal. 2013 Jan;18(1):e168–73. doi: 10.4317/medoral.18324 23229257 PMC3548639

[14] Klages U, Erbe C, Sandru SD, Brüllman D, Wehrbein H. Psychosocial impact of dental aesthetics in adolescence: validity and reliability of a questionnaire across age-groups. Qual Life Res Int J Qual Life Asp Treat Care Rehabil. 2015 Feb;24(2):379–90. doi: 10.1007/s11136-014-0767-8 25092437

[15] Paredes CL. Multidimensional Ethno-racial Status in Contexts of *Mestizaje*: Ethno-racial Stratification in Contemporary Peru. Socius Sociol Res Dyn World. 2018 Jan 1;4:237802311876200.

[16] Zou G. A Modified Poisson Regression Approach to Prospective Studies with Binary Data. Am J Epidemiol. 2004 Apr 1;159(7):702–6. doi: 10.1093/aje/kwh090 15033648

[17] Barros AJ, Hirakata VN. Alternatives for logistic regression in cross-sectional studies: an empirical comparison of models that directly estimate the prevalence ratio. BMC Med Res Methodol. 2003 Dec;3(1):21. doi: 10.1186/1471-2288-3-21 14567763 PMC521200

[18] Nong P, Raj M, Creary M, Kardia SLR, Platt JE. Patient-Reported Experiences of Discrimination in the US Health Care System. JAMA Netw Open. 2020 Dec 15;3(12):e2029650. doi: 10.1001/jamanetworkopen.2020.29650 33320264 PMC7739133

[19] Hargraves JL, Cosenza C, Elliott MN, Cleary PD. The effect of different sampling and recall periods in the CAHPS Clinician & Group (CG-CAHPS) survey. Health Serv Res. 2019 Oct;54(5):1036–44. doi: 10.1111/1475-6773.13173 31132159 PMC6736918

[20] Kjellsson G, Clarke P, Gerdtham UG. Forgetting to remember or remembering to forget: A study of the recall period length in health care survey questions. J Health Econ. 2014 May;35:34–46. doi: 10.1016/j.jhealeco.2014.01.007 24595066

[21] Lewis TT, Cogburn CD, Williams DR. Self-Reported Experiences of Discrimination and Health: Scientific Advances, Ongoing Controversies, and Emerging Issues. Annu Rev Clin Psychol. 2015 Mar 28;11(1):407–40. 25581238 10.1146/annurev-clinpsy-032814-112728 PMC5555118

[22] Grollman EA, Hagiwara N. Measuring Self-Reported Discrimination: Trends in Question Wording Used in Publicly Accessible Datasets. Soc Curr. 2017 Aug;4(4):287–305.

[23] Sischo L, Broder HL. Oral Health-related Quality of Life. J Dent Res. 2011 Nov;90(11):1264–70. doi: 10.1177/0022034511399918 21422477 PMC3318061

[24] Sampson A, Jeremiah HG, Andiappan M, Newton JT. The effect of viewing idealised smile images versus nature images via social media on immediate facial satisfaction in young adults: A randomised controlled trial. J Orthod. 2020 Mar;47(1):55–64. doi: 10.1177/1465312519899664 32031041

[25] Klages U. Dental aesthetics, self-awareness, and oral health-related quality of life in young adults. Eur J Orthod. 2004 Oct 1;26(5):507–14. doi: 10.1093/ejo/26.5.507 15536839

[26] Phelan JC, Link BG. Is Racism a Fundamental Cause of Inequalities in Health? Annu Rev Sociol. 2015 Aug 14;41:311–30.

[27] Pérez DJ, Fortuna L, Alegria M. Prevalence and Correlates of Everyday Discrimination among U.S. Latinos. J Community Psychol. 2008 May 1;36(4):421–33. doi: 10.1002/jcop.20221 19960098 PMC2786077

